# Strongyloidiasis: The Cause of Multiple Gastrointestinal Ulcers in an Immunocompetent Individual

**DOI:** 10.1155/2014/346256

**Published:** 2014-02-04

**Authors:** Shail Sheth, Fady Asslo, Rabih Hallit, Raymund Sison, Muhammad Afridi, Robert Spira, Joseph DePasquale, Jihad Slim, Jack Boghossian

**Affiliations:** ^1^Department of Internal Medicine, Saint Michael's Medical Center, Newark, NJ 07102, USA; ^2^Department of Infectious Disease, Saint Michael's Medical Center, Newark, NJ 07102, USA; ^3^Department of Gastroenterology, Saint Michael's Medical Center, Newark, NJ 07102, USA

## Abstract

Strongyloidiasis is a common parasitic disease in tropical regions of the world. Infection with *Strongyloides stercoralis* usually remains asymptomatic with peripheral eosinophilia and uncontrolled growth. Consequently, immunocompromised individuals are at a higher risk of complications of this disease. We present a case of an immunocompetent patient whose complaint of acute abdominal pain was found to be due to gastric and duodenal ulcerations. Laboratory examination revealed significantly elevated absolute eosinophil count at 11,466/mm^3^ (normal 0–700/mm^3^). The duodenal biopsy revealed parasitic ova and adult worms suggestive of *Strongyloides stercoralis* nematode with increased eosinophils in the tissue. We report the first case of multiple gastric and duodenal ulcerations due to *Strongyloides stercoralis* in an immunocompetent patient. We suggest that the elevated eosinophil count played a central role in the pathogenesis.

## 1. Introduction

Global prevalence of *Strongyloides stercoralis* infection is unknown, but it is estimated that more than three million people are infected worldwide. Infected patients usually remain asymptomatic with peripheral eosinophilia or may complain of myriad of symptoms including skin rash due to larval penetration, cough, wheezing, dyspnea, upper abdominal pain, nausea, vomiting, or diarrhea. *Strongyloides stercoralis* induced gastrointestinal ulcer disease in immunocompromised patients has been well described in the literature but there is only one reported case of a gastric ulcer occurring in an immunocompetent individual due to *Strongyloides stercoralis* infection.

## 2. Case Report

**Figure 1:**
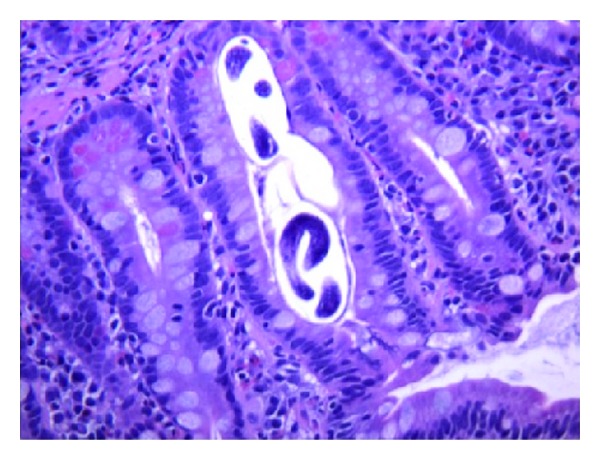
Duodenal mucosa showing adult worms of *Strongyloides stercoralis* associated with chronic duodenitis and increased eosinophils (Hematoxylin and eosin stain, high power field image).

An 83-year-old male born in Dominican Republic with a past medical history of arterial hypertension and asthma presented with complaint of constant abdominal pain for 5 days prior to admission, located in the epigastric area. The patient denied having any fever, chills, nausea, emesis, or altered bowel movements. Additionally, the patient denied using any inhaled corticosteroids or any other medications. He had recently returned from a trip to the Dominican Republic one week prior to his present admission. Not being able to recall any sick contacts, the patient on physical examination was afebrile with a mildly elevated blood pressure of 142/79 mmHg and a soft abdomen without any tenderness. Initially laboratory examination was remarkable for an elevated white cell count of 18,200/mm^3^ (normal 4,500–11,000/mm^3^) with a markedly elevated absolute eosinophil count of 11,466/mm^3^ (normal 0–700/mm^3^), while the rest of his laboratory studies were unremarkable. A computed tomography scan of the abdomen was performed and was also unremarkable. Upper endoscopy showed multiple ulcers in the antrum and the bulb of the duodenum. On the biopsy, parasitic ova and adult worms suggestive of *Strongyloides stercoralis* with a high number of eosinophils were seen by the pathologist (Figure 1). The patient was then placed on albendazole which significantly improved his peripheral eosinophilia to a normal level.

## 3. Discussion

Upper gastrointestinal ulcer due to *Strongyloides stercoralis* infection is a rare entity in immunocompetent patients with only one case reported. Patients with a compromised immune system are predisposed to disseminated disease that involves multiple systems with subsequent possible septic shock. Patients with a history of human immunodeficiency virus (HIV) or human T-lymphotropic virus (HTLV-1) infection, malignancy, current chemotherapy, corticosteroid use, malnutrition, chronic pulmonary diseases, diabetes mellitus, or alcoholism are at a high risk of disseminated *Strongyloides stercoralis* [1] due to several mechanisms [1, 2] which involve immunosuppression of eosinophilic response and lymphocytic activation against the intestinal helminth in combination with an altered intestinal motility rate to create a nurturing environment for *Strongyloides stercoralis* to mature into an adult worm and invade the mucosal barriers of the gastrointestinal tract. The immune response against the helminthic infestation is mainly controlled by the lymphocytes, namely, the T-cell helper type 2 lymphocytes with CD4 markers (TH2 cells), that secrete important cytokines, especially Interleukin-4 (IL-4), Interleukin-5 (IL-5), and Interleukin-10 (IL-10) in response to the helminthic exposure [2]. IL-4 induces an inflammatory process that promotes mast cell recruitment and intestinal goblet cell activation which alters gut physiology, ultimately dislodging the worm [3]. IL-5 plays a major role in the differentiation and maturation of the eosinophil, thus increasing eosinophil counts. The eosinophils carry toxic granules which contain major basic protein (MBP), eosinophil cationic protein (ECP), eosinophil derived neurotoxin (EDN), and eosinophil peroxidase (EPO) which are directly toxic to the larvae of *Strongyloides stercoralis* [3, 4]. ECP and EDN possess ribonuclease activity that form pores into the membrane of target cells, facilitating the entry of other toxic molecules into the cells with subsequent degeneration. Unfortunately, these toxic granules have cytotoxic effects on the gastrointestinal epithelium and may result in ulcer formation [4].

High eosinophil counts in the serum should prompt the provider to screen for parasitic disease. If there is still a strong suspicion, the absence of eosinophilia is not sensitive enough to rule out helminthic infections due to the fact that the eosinophils are mainly tissue dwelling cells [3, 4]. Eosinophils are more numerous in tissue, a hundredfold more than in the peripheral blood. They are seen in body surfaces that have direct interaction with the environment like the respiratory tract, gastrointestinal tract (except esophagus), and lower genitourinary tract [3]. In a study by Loutfy et al. [5], sixty-nine of seventy-six patients positive for *Strongyloides stercoralis* as diagnosed by stool tests in total had peripheral eosinophilia. In this pool of the patients, the highest eosinophil count was 3310 eosinophil/mm^3^ and the median was 740 eosinophil/mm^3^ (9-10% of the total white cell count). Absolute eosinophil count in the serum has been graded as mild (500/mm^3^ to 1,500/mm^3^), moderate (1,500/mm^3^ to 5,000/mm^3^), and severe (more than 5,000/mm^3^). Usually moderate eosinophilia is required before tissue damage occurs; however there is no reliable level that precisely reflects the concentration of the activated eosinophils within the affected tissue. Our patient had total white blood cell (WBC) count of 18,200/mm^3^ and the manually calculated eosinophil count was 11,466/mm^3^, accounting for sixty-three percent of all WBCs, which is the highest reported number in the literature in all strongyloidiasis cases. We suggest that *Strongyloides stercoralis* nematode infection induced a dramatic local inflammatory response in our immunocompetent patient as indicated by absolute value of eosinophil cells. This extremely high number of eosinophils as a result released an increased amount of toxic granules that produced multiple ulcerations in the upper gastrointestinal tract. We report the first case of multiple gastric and duodenal ulcers due to *Strongyloides stercoralis* infection in an immunocompetent patient with markedly elevated eosinophil cell counts and we suggest that the eosinophil cells played a central role in the development of the ulcers.
